# Ultra-low HIV-1 p24 detection limits with a bioelectronic sensor

**DOI:** 10.1007/s00216-019-02319-7

**Published:** 2019-12-21

**Authors:** Eleonora Macchia, Lucia Sarcina, Rosaria Anna Picca, Kyriaki Manoli, Cinzia Di Franco, Gaetano Scamarcio, Luisa Torsi

**Affiliations:** 1 Dipartimento di Chimica, Università degli Studi di Bari Aldo Moro, Via E. Orabona 4, 70125 Bari, Italy; 2 The Faculty of Science and Engineering, Åbo Akademi University, Porthaninkatu 3, FI-20500 Turku, Finland; 3 Dipartimento Interateneo di Fisica “M. Merlin”, Università degli Studi di Bari Aldo Moro, Via E. Orabona 4, 70125 Bari, Italy; 4 Unità di Bari, CNR - Istituto di Fotonica e Nanotecnologie, Via E. Orabona 4, 70125 Bari, Italy; 5 CSGI (Centre for Colloid and Surface Science), Via E. Orabona 4, 70125 Bari, Italy

**Keywords:** Organic bioelectronics, Electrolyte-gated thin-film transistors, Single molecule detection with a transistor (SiMoT), HIV-1 p24 detection

## Abstract

Early diagnosis of the infection caused by human immunodeficiency virus type-1 (HIV-1) is vital to achieve efficient therapeutic treatment and limit the disease spreading when the viremia is at its highest level. To this end, a point-of-care HIV-1 detection carried out with label-free, low-cost, and ultra-sensitive screening technologies would be of great relevance. Herein, a label-free single molecule detection of HIV-1 p24 capsid protein with a large (*wide-field*) single-molecule transistor (SiMoT) sensor is proposed. The system is based on an electrolyte-gated field-effect transistor whose gate is bio-functionalized with the antibody against the HIV-1 p24 capsid protein. The device exhibits a limit of detection of a single protein and a limit of quantification in the 10 molecule range. This study paves the way for a low-cost technology that can quantify, with single-molecule precision, the transition of a biological organism from being “healthy” to being “diseased” by tracking a target biomarker. This can open to the possibility of performing the earliest possible diagnosis.

**Electronic supplementary material:**

The online version of this article (10.1007/s00216-019-02319-7) contains supplementary material, which is available to authorized users.

## Introduction

Bioelectronic label-free sensors, based on an electrolyte-gated organic field-effect transistor [1, 2], endowed with selectivity via the integration of suitable bio-recognition elements, have lately exhibited sensitivity down to the physical limit [3–8]. The key to single-molecule detection with a transistor, SiMoT [3], is the immobilization of trillions of capturing antibodies on a millimetre-size gate. The extremely large number of capturing sites covalently attached to a large interface, addressed as wide-field sensing approach [9], increases the interaction cross-section with the biomarker to be detected, allowing the assay of extremely diluted solutions. At the same time, a cooperative effect, enabled by a hydrogen-bonding network and fuelled by the transistor operating gate voltage, amplifies the impact of a single or of few bindings that can be detected as a sizable variation of the transistor channel current [3, 6]. The SiMoT platform has been also shown capable of selective detection of a single protein in real fluids such as diluted saliva and of 15 proteins in whole serum [3]. This disruptive new technology that is also label-free offers a completely novel approach to biosensing compared with state-of-the-art label-free single-molecule sensing based on nanoscale devices [8]. Differently from the elicited nanotechnology-based systems, the SiMoT platform is fabricated via solution-processed, scalable procedure and it is foreseen that it will be further developed into a low-cost large-area printed technology [1]. This makes SiMoT extremely promising as a disposable point-of-care (POC) system for early diagnostics.

POC early diagnosis of the infection caused by human immunodeficiency virus type-1 (HIV-1) is vital to achieve efficient therapeutic treatment and to limit the disease spreading when the viremia is at its highest level. The World Health Organization has estimated that 37.9 million people were infected globally with human immunodeficiency virus (HIV) at the end of 2018, the majority of them in developing countries [10]. Early detection of HIV-1 infection plays a key role on the prompt treatment of primary HIV-1 infection, being the way to achieve a more effective immune response to the virus, also preventing vulnerability to opportunistic infections. On the other hand, early detection of HIV-1 infection may have a terrific impact on the public health level as well, reducing the risk of unknowing spread of HIV-1 during acute infection [11, 12]. The current risk of HIV-1 transmission from screened blood has been estimated to range from 1 in 38,000 to 1 in 153,000 [13, 14]. In fact, HIV-1 virus is a capsule composed of proteins such as the p24 that are referred to as antigens because they induce an immune response in the infected host [15]. The disease acute phase starts as early as few days after the contraction of the infection. At this stage, both the viral nucleic acid and the HIV-1 p24 antigen are found in the host serum, while the host developed antibodies appear only several months later. The clinical symptoms associated with the developed immunodeficiency become evident even later. The advantage of the HIV-1 virus early detection and the infection prompt diagnosis is, hence, two-fold [16, 17]. The early treatment with antiretroviral therapy of the primary HIV-1 infection enables a more effective immune response. At the societal level, the HIV infection early detection can dramatically impact on the public health management, reducing the risk of an uncontrolled spread of the virus particularly during the acute infection phase.

The HIV diagnostic tests rely on the detection of the host antibodies, the whole virus as well as virus components such as the HIV-1 p24 antigen or the HIV-1 RNA [18]. Indeed, the antigen and the RNA-based platforms are the ones that enable detections in the first acute phase. The polymerase chain reaction of HIV-1 RNA in plasma is the assay that has provided so far the highest performance level [19, 20]. However, the impact is hampered by the need for costly equipment and reagents, hardly affordable where financial resources for healthcare are limited. Besides, since too many false negative results have been observed in patients with only a few copies of HIV-1 RNA [21], the US Food and Drug Administration does not currently approve detection of HIV-1 plasma RNA by polymerase chain reaction [22]. At variance, the HIV-1 p24 virus capsid antigen can be detected; yet, the limit of detection (LOD) of the golden standard platform for protein assay, the enzyme-linked immunosorbent assay (ELISA), is rather high falling in the range of 40 nanomolar (nM, 10^−9^ M) – 0.4 pM (10^−12^ M) [23, 24]. (Table 1) ELISA label-based approaches have been significantly improved with the use of gold nanoparticles [23, 25] or DNA bio-barcodes [26], so as the LOD has been pushed down to 100 fM (10^−15^ M). Even better results have been reached with a molecularly imprinted polymer-based electrochemical sensor that has enabled HIV-1 p24 detection down to a LOD of 3 fM [27]. However, the host serum in the first acute infected phase contains from 10 to 3 × 10^4^ virions per millilitre, resulting in an estimated concentration of HIV-1 p24 ranging from 50 aM (10^−18^ M) to 15 fM [24]. Hence, in a standard 100 μl volume of serum, as low as thousands of HIV-1 p24 antigens can be present.

The aim of the present work is to demonstrate that the SiMoT sensor can detect the HIV-1 p24 protein at a limit of detection (LOD, S/*N* = 3)^2^ of 30 × 10^−21^ M (zM) where 2 ± 1 proteins are found in a 100 μl volume, and at a limit of quantification (LOQ, S/*N* = 10)^2^ of 100 zM, opening to future developments in ultrasensitive POC quantitative testing for early diagnosis of HIV-1 infection.

## Materials and methods

### Materials

Poly(3-hexylthiophene-2,5-diyl), P3HT, regioregularity > 99%, with an average molecular weight of 17.5 kDa (g mol^−1^), was purchased from Sigma-Aldrich and used as field-effect channel material with no further purification. 3-Mercaptopropionic acid (3-MPA), 11-mercaptoundecanoic acid (11-MUA), 1-ethyl-3-(3-dimethylaminopropyl)-carbodiimide (EDC), N-hydroxysulfosuccinimide sodium salt (sulfo-NHS), and 2-propanol were purchased from Sigma-Aldrich and used with no further purification. The anti-HIV-1 p24 produced in mouse is a monoclonal antibody and was purchased from Abcam (Cambridge, UK). The recombinant HIV-1 p24 capsid protein (~ 26 kDa), from Abcam, is produced by *Escherichia coli*. Bovine serum albumin (BSA, molecular weight 66 kDa) was purchased from Sigma-Aldrich and readily used. Water (HPLC-grade, Sigma-Aldrich) and ethanol grade puriss p.a. assay ≥ 99.8% were used with no further purification. Phosphate-buffered saline (PBS, Sigma-Aldrich) solution presents osmolality and ion concentrations, which match those of the human body (isotonic). One tablet of PBS has been dissolved in 200 mL of HPLC-grade water, yielding 0.01 M phosphate buffer, 0.0027 M potassium chloride and 0.137 M sodium chloride, pH 7.4, at 25 °C.

### Electrolyte-gated field-effect transistor fabrication

The transistors were fabricated starting from a silicon substrate covered by thermally grown SiO_2_. Prior to any processing, the SiO_2_ surface was cleaned through a procedure involving sonication in solvents of increasing polarity. Source (S) and drain (D) interdigitated electrodes were defined on the Si/SiO_2_ substrate by using photolithography and electron-beam evaporation of a 5-nm-thick Ti adhesion layer followed by a 50 –nm-thick Au layer. The spacing between S-D fingers (channel length, L) and the total fingers length (channel width, W) are L = 5 μm and W = 7650 μm, respectively. A P3HT solution (2.6 mg ml^−1^ in chlorobenzene) filtered through a 0.2-μm filter was spin-coated at 2 × 10^3^ r.p.m. for 20 s and annealed at 90 °C for 15 min. A polydimethylsiloxane well was glued on the substrate to include the interdigitated channel area and filled with 300 μl of water (HPLC-grade) acting as gating medium. An e-beam-evaporated Ti/Au (5/50 nm) layer on a Kapton® foil (area of ~ 0.6 cm^2^) served as the gate (G) electrode. The gate was stably positioned on the water on top of the well at a distance of about 4 mm from the electrode interdigitated area.

### Gate bio-functionalization protocol

The bio-functionalization procedure involves the covalent attachment to the gate gold surface of a chemical self-assembled monolayer (SAM) to whom the anti-HIV-1 p24 antibodies are conjugated. Before use, the gate electrodes were cleaned in an ultrasonic bath of 2-propanol for 10 min and treated for 10 min in an ozone cleaner. The chemical SAM (chem-SAM) functionalization protocol on the gold surface involved a 10 mM solution of 10:1 molar ratio of 3-MPA to 11-MUA in ethanol. The cleaned gold surface was immersed in the 3-MPA and 11-MUA solution and kept in the dark under constant N_2_ flux for 18 h at 22 °C [28]. The carboxylic groups were activated afterward in a 200 mM EDC and 50 mM sulfo-NHS aqueous solution for 2 h at 25 °C. The anti-HIV-1 p24 antibody was then conjugated to the activated COOH sites reacting with the terminal amine groups of the antibody. To this end, the gate has been immersed in an anti-HIV-1 p24 PBS solution for 2 h at 25 °C. The solution comprises 3.6 μM (0.1 mg ml^−1^) of anti-HIV-1 p24 and PBS at a pH of 7.4 and an ionic strength (*i*_*s*_) of 162 mM. Afterwards, the unreacted sulpho-NHS groups were saturated with ethanolamine (1 M in PBS 10 mM) for 1 h at 25 °C. Finally, the bio-functionalized gate was immersed in a 1.5 μM (0.1 mg ml^−1^) BSA solution in PBS 10 mM for 1 h at 25 °C. This is meant to minimize non-specific binding. The layer of anti-HIV-1 p24 attached to the chem-SAM and processed as described above forms the bio-SAM. The chem-SAM plus the bio-SAM forms what is here addressed as SAM. The negative control experiment was performed by means of a gate electrode functionalized with BSA instead of anti-HIV-1 p24.

The coverage of anti-HIV-1 p24 antibody molecules onto gold surface was estimated by surface plasmon resonance (SPR) experiments performed on a BioNavis MP-SPR Navi™ 200-L instrument using Au-coated glass chips functionalized according to the protocol described above.

**Table 1:** Figures of merits of the most relevant analytical methods for HIV-1 p24 detection

	HIV-1 p24 limit of detection	Selectivity	Dynamic range	Label	Time to result
ELISA [23]	400 nM	High	4 orders of magnitude	Label-needing	Long
Nanoparticle-based bio-barcode [23]	4 nM	High	6 orders of magnitude	Label-needing	Long
Immuno-polymerase chain reaction [25]	100 fM	High	3 orders of magnitude	Label-needing	Short (few hours)
Multi-walled carbon nanotube electrochemical sensor [27]	3 fM	High	4 orders of magnitude	Label needing	Short (few hours)
SiMoT	30 zM (2±1 molecules in 100 mL)	High	2 orders of magnitude	Label-free	Short (few hours)

### Sensing measurements

The transistor current-voltage (I-V) curves were measured with a semiconductor parameter analyser equipped with a probe station, in air and at RT (20–22 °C). Before proceeding with the sensing measurements, the source-drain current, I_D_, was stabilized by cycling the measurement of the transfer curve (I_D_ vs. the gate bias V_G_ at V_D_ = − 0.4 V) of the transistor comprising a clean bare gold gate, until the last three current traces overlapped. A functionalized gate was then incubated (at RT and in the dark) for 10 min in 100 μl of PBS. The gate was removed from the PBS solution, washed thoroughly with HPLC water, mounted on the transistor (exactly replacing the bare gold gate previously used) and a new transfer characteristic was registered. After the measurement of the I_0_ base line, the same gate was immersed and incubated for 10 min in 100 μl of the PBS standard solutions of the HIV-1 p24 analyte with nominal concentrations ranging from 1 to 1 × 10^7^ zM. After incubation in each of the PBS standard solutions of HIV-1 p24 starting from the more diluted one, the SAM was washed thoroughly with HPLC water to remove the unreacted ligands and a further I-V transfer curves were measured. All the data points presented in this study are averaged over three replicates. The resulting reproducibility error is computed as the relative standard deviation. Further, three BSA-functionalized gates were used to measure the HIV-1 p24 protein negative control dose curves as well.

## Results and discussion

**Fig. 1:**
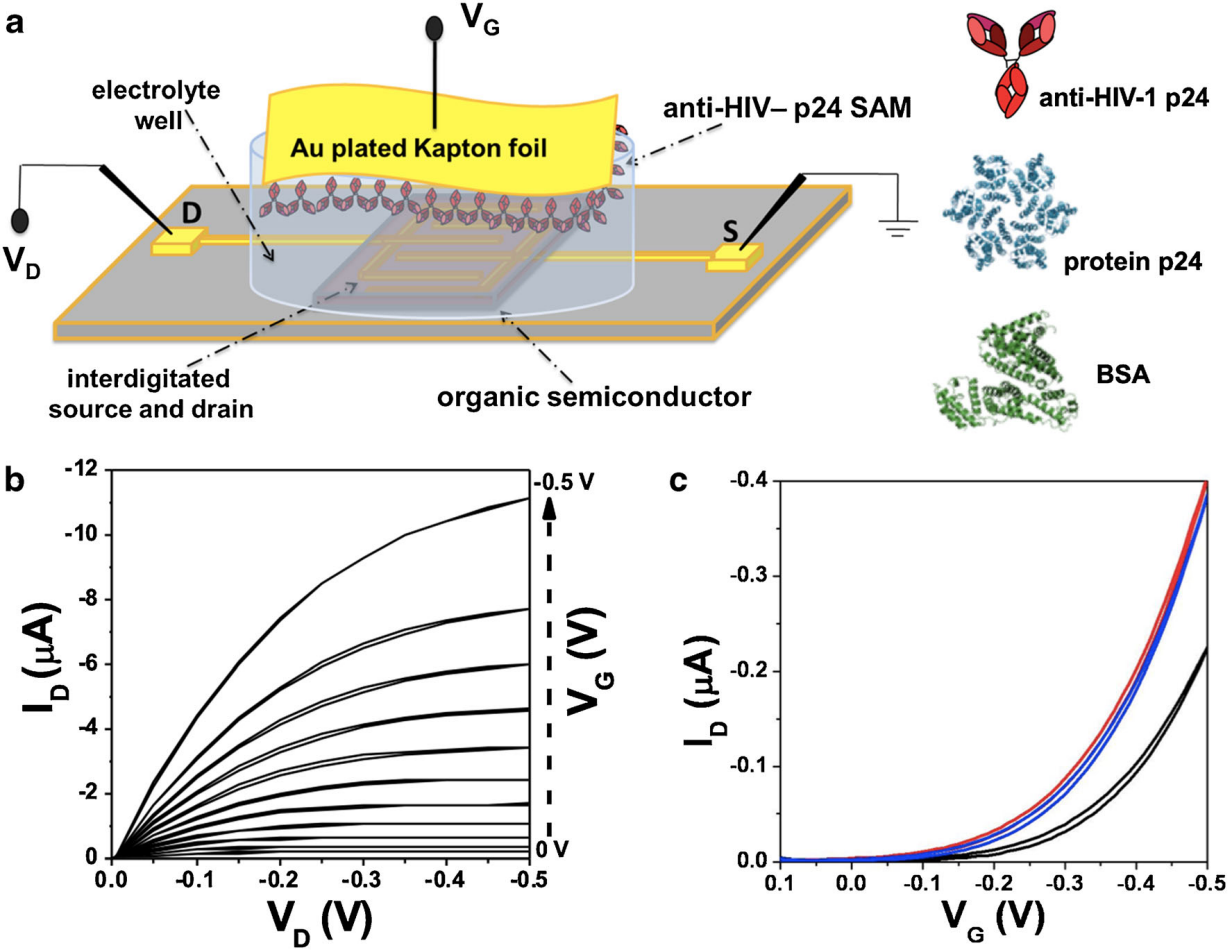
**a** The three-dimensional schematic structure of the electrolyte-gated organic field-effect transistors used for single-molecule detection along with the sketches of the proteins used. **b** Output characteristics (I_D_ vs. V_D_, with V_G_ ranging from 0 to − 0.5 V in steps of − 0.05) measured with a gold-plated Kapton gate. **c** Transfer characteristics (I_D_ vs. V_G_ ranging from 0.1 to − 0.5 V at constant V_D_ = − 0.4 V) for a gold plate gate before and after the measurement of the dose response curve (red and blue line respectively) and for an anti-HIV-1 p24-functionalized gate (black curve)

In Fig. 1 a, a schematic representation of the SiMoT was shown. HPLC-grade deionized water acts as the electrolyte dielectric [29]. Details on the bio-functionalization protocol are reported elsewhere [30] and summarized in the “Materials and methods” section. The efficiency of the overall bio-functionalization process was assessed by spectroscopic surface analysis in a previous study [30]. Moreover, SPR characterization was carried out to evaluate the average coverage (Γ, ng/cm^2^) of the bioreceptor on the gold surface. In Fig. S1 (see Electronic Supplementary Material, ESM), the binding of the antibody to the surface is monitored as a function of time resulting in a Γ = 80 ± 13 ng/cm^2^, calculated from the angular shift as reported in previous works [6, 7]. This value can be converted in the average total number of active binding sites being equal to 1.94 ± 0.03 × 10^12^ cm^−2^ considering the molecular weight of the antibody. This means that a dense layer of anti-HIV-1 p24 molecules is formed over the chem-SAM being capable to support the binding event even in extremely diluted solutions. It is worth mentioning that the amide groups formed by ethanolamine functionalization step originate hydrogen bonds (H-bonds) that connect neighbouring chains. A dipole moment is associated with each H-bond, so taken all-together, the H-bond system results in an electrostatic network that keeps the whole chem-SAM connected. It has been postulated that the elicited network of H-bonds can sustain electrostatic cooperative interactions, responsible for the extremely high sensitivity of the SiMoT [3, 6]. In fact, it has been demonstrated that, once this electrostatic connecting element is removed, the sensing is suppressed. Typical output characteristics measured with a gold-plated Kapton gate, where the drain current I_D_ is measured as a function of drain voltage V_D_ at gate voltages V_G_ ranging from 0 to − 0.5 V in steps of − 0.05 V, are shown Fig. 1b.

The curves exhibit a good level of current modulation as well as nicely shaped linear and saturated regions along with low leakage currents at low V_D_. Moreover, the curves were measured in the forward and reverse mode to prove that a negligible hysteresis is present. The I_D_–V_G_ transfer curves at a fixed V_D_ = − 0.4 V are shown in Fig. 1 c. The red curve is the current measured on a bare gold gate after the stabilization of the SiMoT. A stable current is reached by cycling the device in the 0.1 < V_G_ < − 0.5 V range in water, until three subsequent current traces fully overlap. The blue curve is the current measured on the same SiMoT device with the same gold gate after the measurement of a whole calibration curve. An anti-HIV-1 p24-functionalized gate was used for the HIV-1 p24 protein sensing measurements. The current level with an Au gate was recorded before and after the assay. This control experiment allows assessing that only the dose curves that involve an I_D_ current decrease within 5% are acceptable. Such a procedure rules out that any current changes measured during the sensing experiments could be attributed to the organic semiconductor performance degradation. The black curve is the current measured when the gate is functionalized with anti-HIV-1 p24. A shift of the threshold voltage (V_T_) suggests a change in the gate electrochemical potential or a work function change after the surface functionalization protocol. This shift is connected with the presence of a net dipole moment on the gate surface [3]. In this case, a threshold voltage V_T_ of − 0.20 + 0.05 V was observed with the anti-HIV-1 p24-functionalized gate, while a V_T_ of − 0.12 + 0.05 V was measured with the bare gold gate.

**Fig. 2:**
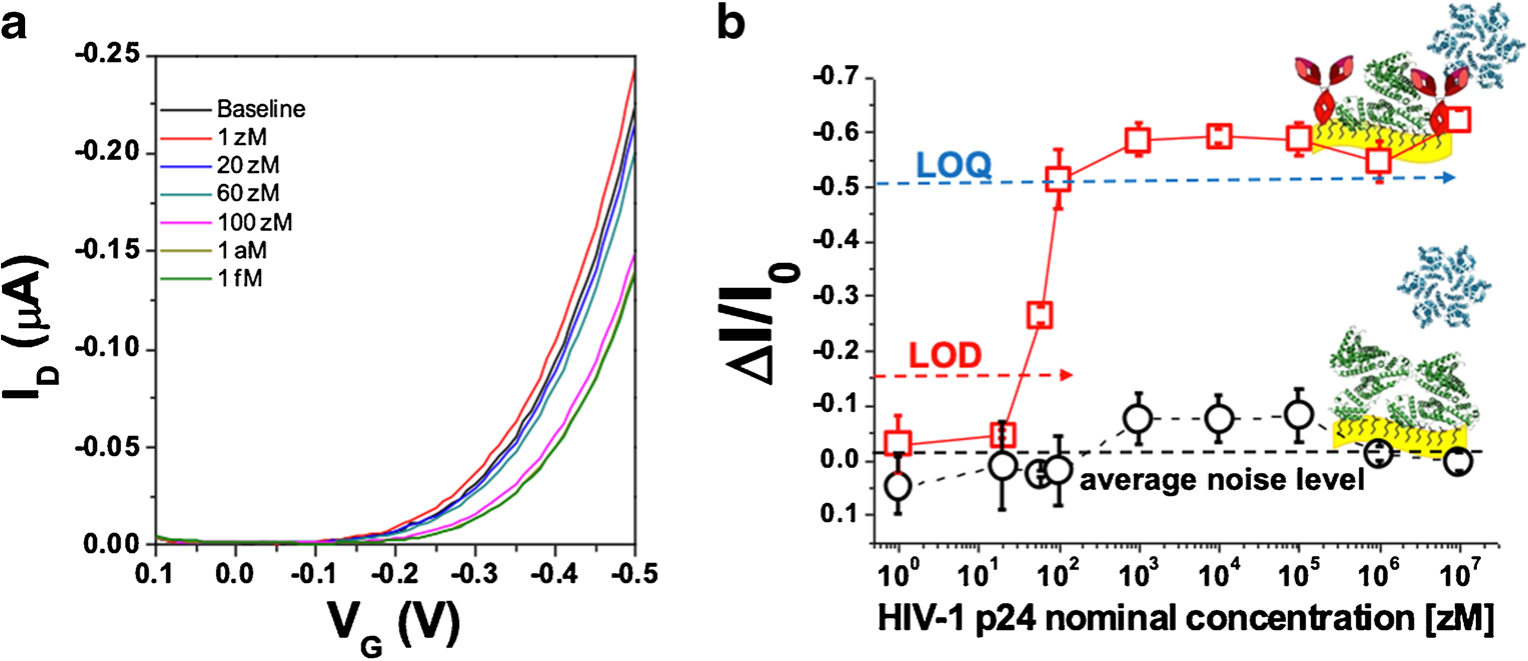
**a** SiMoT transfer characteristics (I_D_ vs. V_G_ at V_D_ = − 0.4 V). The black curve (barely visible because falling under the blue one) corresponds to the anti-HIV-1 p24-functionalized gate incubated in the bare PBS solution. The same gate is further exposed, in sequence, to PBS standard solutions of HIV-1 p24 antigen at concentrations of 1 zM (red curve), 20 zM (blue curve), 60 zM (dark cyan curve), 100 zM (magenta curve), 1 × 10^3^ zM (dark yellow curve), and 1 × 10^6^ zM (olive curve). **b** HIV-1 p24/anti-HIV-1 p24 affinity binding calibration curve (red squares) as the relative change of the I_D_ current (*ΔI*/*I*_*o*_ see text) vs. the HIV-1 p24 concentration. The black circles are the negative control responses of the bare BSA-functionalized gate to HIV-1 p24 solutions. The proteins are assayed from standard solutions in PBS. Data are relevant to an ensemble of measurements acquired on two different devices (reproducibility error) and are reported as the average value along with the relevant relative standard deviations

The HIV-1 p24 protein sensing was performed by measuring the SiMoT transfer characteristics after incubation of the anti-HIV-1 p24 SAM for 10 min in 100 μl of PBS. A stable I_0_*baseline* (black line in Fig. 2a) was eventually recorded. Then, the same gate was immersed and incubated for 10 min in 100 μl of the PBS standard solutions of the marker (HIV-1 p24 protein) with nominal concentrations ranging from 1 to 1 × 10^7^ zM, and the relevant transfer curves were recorded. The PBS solution reproduces a physiologically relevant fluid with a pH of 7.4 and ionic strength of 162 mM, mimicking the environment of blood serum. After each incubation step, the bio-functionalized gate electrode was washed with water to remove the unreacted ligands and new I-V transfer curves were measured.

The 1 zM curve in Fig. 2 a shows no change compared with the baseline as, according to Poisson sampling, in 100 μl at 1 zM, no ligand is present. In fact, the nominal number of ligand (#p24) at each concentration in an incubation volume of 100 μl can be evaluated according to the following equation

1 $$ \#p24= cV{N}_A $$

where *c* is the HIV-1 p24 concentration, *V* is the incubation volume and *N*_*A*_ the Avogadro’s number. No significant changes compared with the baseline have been observed at 20 zM as well, where Poisson sampling foresees that 1 ± 1 particle can be found in 100 μl. According to Poisson statistics, the error bar is taken as the square root of the estimated number of particles. The light green curve, measured at 60 zM concentration shows a significant current decrease as well as a shift towards more negative gate potentials. A current change is expected; as in this case, 4 ± 2 ligands are found in 100 μl. In fact, at least 2 ligands are always present in the sampled volume in 100 μl at 60 zM. A further current decrease was measured at a concentration of 100 zM, where 6 ± 2 ligands are present, reaching a saturation value. The analysis of the transfer curves showed that, upon binding, a shift of V_T_ towards more negative values occurs. This has been attributed to a variation of the surface dipole moment possibly due to a change in the H-bond structure [3, 6].

The relative current change upon exposure of the anti-HIV-1 p24 SAM to the ligands in the PBS solutions, *ΔI*/*I*_*0*_ = [(*I*-*I*_*0*_)/*I*_*0*_], has been used as the sensing response, where the *I* current values at each concentration are taken from the relevant transfer curves (Fig. 2a) at the V_G_ that maximises the trans-conductance. The *ΔI/I*_*0*_ vs. HIV-1 p24 protein concentration dose curve measured with the anti-HIV-1 p24-functionalized gate is shown in Fig. 2 b as red squares, while the black circles are the negative control responses measured exposing the BSA-functionalized gate to HIV-1 p24 proteins. This to demonstrate that the measured SiMoT response to HIV-1 p24 is selective as it is largely ascribable to the presence of the target analyte in the investigated sample. The error bars are taken as one standard deviation. The limit of detection (LOD) level was evaluated as the concentration providing a response equal to the average of the noise level of the negative control experiment in the whole concentration range plus three times the noise standard deviation [2]. The LOD level for the HIV-1 p24 sensing with the anti-HIV-1 p24 SAM is 16% that corresponds to a concentration of about 30 zM. The number of HIV-1 p24 proteins at the LOD is equal to [c] × *V* × *N*_*A*_, where [*c*] = 30 × 10^−21^ mol l^−1^, *V* = 100 μl and *N*_*A*_ = Avogadro’s number; this corresponds to a LOD of 2 ± 1 molecules. For the limit of quantification (LOQ), ten times the noise standard deviation was considered. This assures that, above this limit, both the false negative and the false positive are below 1%. This corresponds to a response of 47% and a concentration of 100 zM, which turns into 10 ± 3 molecules in the usual volume.

The sensing mechanism that enabled such a low detection limit was addressed in detail elsewhere [3, 6] postulating that a hydrogen bonding network (H-bond) characterizing the chem-SAM was involved. As anticipated, after the conjugation of the capturing antibodies (anti-HIV-1 p24) to the activated chem-SAM, ethanolamine was added to block the activated carboxylic groups that were not activated. The amide groups that are formed originate hydrogen bondings connecting the oxygen of the amide group in one chain to the hydrogen of the amide group of the neighbouring one. A dipole moment is associated to each H-bond; hence, an electrostatic network is generated. The SiMoT field-effect transduction is sensitive to surface dipoles on the gate and to their changes. Indeed, the single binding occurs involving just one antibody that undergoes a conformational change that changes only locally the dipole moment on the gate. However, molecular simulation [3] showed that, due to the extremely large number of carboxylic chains connected via the H-bond network as well as of the large number of antibodies that are packed at the physical limit (10^4^ per μm^2^) on the gate surface, the localized conformational and electrostatic change propagates as an electrostatic change fuelled by the measuring gating field. In that, the single binding event is assumed to generate a defect in the H-bond network that locally changes the gate surface dipole moment. This change propagates once the network in immersed in the gating field to perform the measurement. This electrostatic variation is hence a cooperative effect that involves a large number of antibodies and so it amplifies the response of the single binding event, making the field-effect measurement possible [3, 6]. The model foresees also that saturation is reached when an extremely large number of antibodies are involved in the cooperative response [3]. In particular, according to this model, the SAM is conceptualized as an ensemble of domains comprising a given number of capturing antibodies. The binding of one single ligand to the capturing antibody of a given domain produces a change of its work function, upon collaborative interactions propagating the change. The model foresees that this process is irreversible. Thus, any other binding events taking place within the same domain is not capable to produce further changes in the domain work function. Besides, the more compact and electrostatically connected the SAM is, the larger the domain generated and hence the steeper the dose curve in the zM range is. In particular, ligand concentrations close to the affinity of the antigen-antibody system, falling in the nM range as reported in Abcam (Cambridge, UK) datasheets, have been investigated elsewhere [3], showing that the SiMoT platform does not allow to gather any information at higher concentrations, being the electrostatically driven transduction mechanism limited by the dimension of the work function domains.

The world record ultra-low detection and quantification limits make the SiMoT platform extremely interesting for POC diagnostics of biomarkers of progressive diseases as it would potentially enable an unprecedented early diagnostic. When it comes to HIV-1, the SiMoT technology could give hope for the detection of the HIV-1 virus in the first acute phase of the infection.

## Conclusions

In summary, a SiMoT sensor operated with a gate electrode functionalized with anti-HIV-1 p24 antibody SAM has been developed for the detection of the HIV-1 p24 antigen at the single molecule limit. An unprecedented detection limit of 30 zM, corresponding to a LOD of 2 ± 1 molecules, was demonstrated. Therefore, the biosensing platform herein proposed could suit POC applications to enable the diagnosis of the HIV-1 infection in the very first stage, namely as early as few days after infection. In fact, the proposed biosensing platform opens up new relevant opportunities in the field of ultrasensitive point-of-care testing for early diagnosis of HIV-1 infection, with an unpreceded impact on the public health level, reducing the risk of unknowing spread of HIV-1 during acute infection. Importantly, the biosensing platform herein proposed is in principle applicable for the detection of a wide spectrum of clinically relevant biomarkers.

## Electronic supplementary material

ESM 1 (PDF 111 kb)

## Abbreviations

SiMoT: Single-molecule with a transistor
POC: Point-of-care
LOD: Limit of detection
LOQ: Limit of quantification
SAM: Self-assembled monolayer
