# Supplementary material for: Ultra-low HIV-1 p24 detection limits with a bioelectronic sensor
Source: Anal Bioanal Chem. 2019 Dec 21;412(4):811–8. doi: 10.1007/s00216-019-02319-7 (PMC7005089; doi:10.1007/s00216-019-02319-7)
Supplement: Supplementary file 1 — (PDF 111 kb) [file 216_2019_2319_MOESM1_ESM.pdf]

Analytical and Bioanalytical Chemistry

Electronic Supplementary Material

Ultra-low HIV-1 p24 detection limits with a bioelectronic sensor

Eleonora Macchia, Lucia Sarcina, Rosaria Anna Picca, Kyriaki Manoli, Cinzia Di Franco, Gaetano Scamarcio, Luisa Torsi

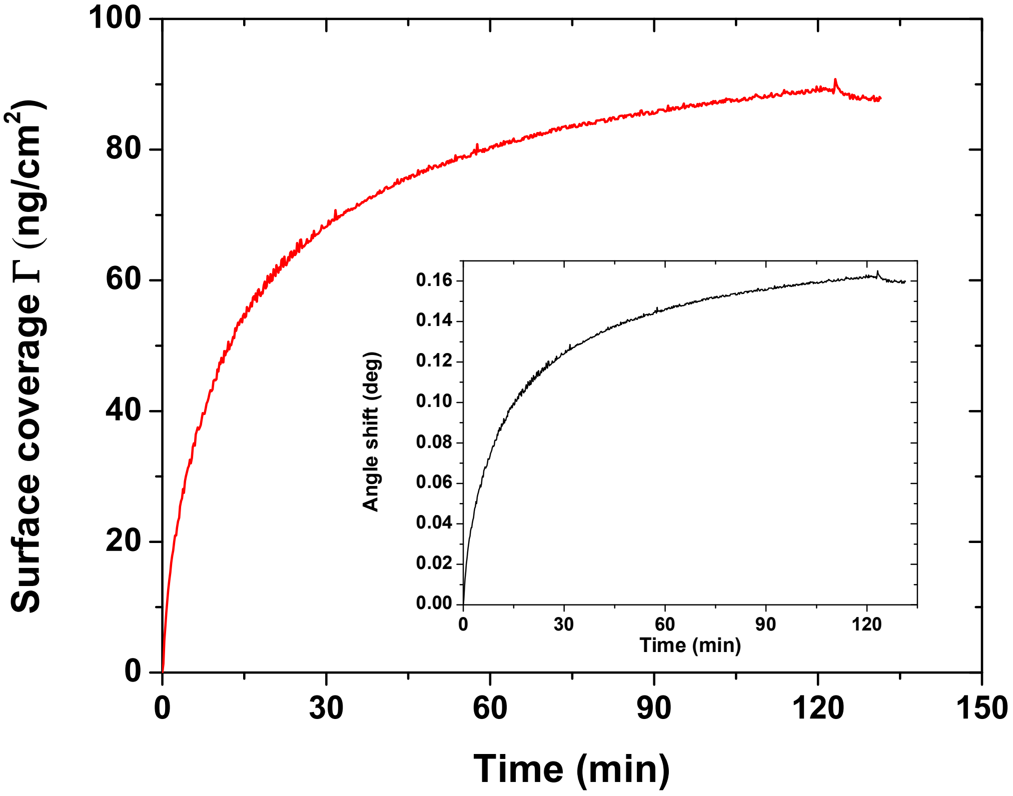

Fig. S1 Anti-HIV p-24 surface loading vs. time. Inset: corresponding angular response of the bioreceptor binding to the surface as a function of time
